# The Vinyasa Tool for mHealth Solutions: Supporting Human-Centered Design in Nascent Digital Health Ecosystems

**DOI:** 10.2196/45250

**Published:** 2023-10-02

**Authors:** Verghese Thomas, Bharat Kalidindi, Abijeet Waghmare, Abhishek Bhatia, Tony Raj, Satchit Balsari

**Affiliations:** 1 Division of Medical Informatics St John's Research Institute Bangalore India; 2 Carolina Health Informatics Program University of North Carolina at Chapel Hill Chapel Hill, NC United States; 3 Department of Physiology St John's Medical College Bangalore India; 4 Department of Emergency Medicine Beth Israel Deaconess Medical Center Harvard Medical School Boston, MA United States; 5 François-Xavier Bagnoud Center for Health and Human Rights Harvard T.H. Chan School of Public Health Boston, MA United States

**Keywords:** community health workers, digital health, focus group discussions, health care workers, human centered design, key informant interviews, LMICs, low- and middle-income countries, mHealth, mobile health, qualitative research

## Abstract

**Background:**

mHealth (mobile health) systems have been deployed widely in low- and middle-income countries (LMICs) for health system strengthening, requiring considerable resource allocation. However, most solutions have not achieved scale or sustainability. Poor usability and failure to address perceived needs are among the principal reasons mHealth systems fail to achieve acceptance and adoption by health care workers. A human-centered design approach to improving mHealth system use requires an exploration of users’ perceptions of mHealth systems, including the environmental, user-related, and technological aspects of a system. At present, there is a dearth of contextually intelligent tools available to mHealth developers that can guide such exploration before full-scale development and deployment.

**Objective:**

To develop a tool to aid optimization of mHealth solutions in LMICs to facilitate human-centered design and, consequently, successful adoption.

**Methods:**

We collated findings and themes from key qualitative studies on mHealth deployment in LMICs. We then used the Informatics Stack framework by Lehmann to label, sort, and collate findings and themes into a list of questions that explore the environment, users, artifacts, information governance, and interoperability of mHealth systems deployed in LMICs.

**Results:**

We developed the Vinyasa Tool to aid qualitative research about the need and usability of mHealth solutions in LMICs. The tool is a guide for focus group discussions and key informant interviews with community-based health care workers and primary care medical personnel who use or are expected to use proposed mHealth solutions. The tool consists of 71 questions organized in 11 sections that unpack and explore multiple aspects of mHealth systems from the perspectives of their users. These include the wider world and organization in which an mHealth solution is deployed; the roles, functions, workflow, and adoption behavior of a system’s users; the security, privacy, and interoperability afforded by a system; and the artifacts of an information system—the data, information, knowledge, algorithms, and technology that constitute the system. The tool can be deployed in whole or in part, depending on the context of the study.

**Conclusions:**

The Vinyasa Tool is the first such comprehensive qualitative research instrument incorporating questions contextualized to the LMIC setting. We expect it to find wide application among mHealth developers, health system administrators, and researchers developing and deploying mHealth tools for use by patients, providers, and administrators. The tool is expected to guide users toward human-centered design with the goal of improving relevance, usability, and, therefore, adoption.

## Introduction

In this paper, we present a novel framework for conducting human-centered design evaluations of mobile health (mHealth) tools used by physician and nonphysician clinical health care workers. We describe the development of our framework, the Vinyasa Tool, aimed at guiding qualitative research on mHealth design and implementation, particularly in low- and middle-income countries (LMICs), grounded in the principles of human-centered design.

Governments across the world have implemented mHealth systems that integrate mobile technology into health care delivery to strengthen health systems [1]. These systems use mobile technology to support public health and clinical practice [2]. Implementations of mHealth have been increasing among LMICs as health systems seek to use expanding telecommunication networks to improve public health system access and processes [3,4]. However, despite large investments, few mHealth solutions in LMICs have succeeded in scaling up or attaining sustainability [5]. There is little evidence that mHealth interventions have substantively strengthened health systems [6-8].

Limited usability of digital health tools has been identified as a significant uptake barrier among health care workers and other mHealth system users that must be overcome for successful digital health implementation [9,10]. Usability has been defined as “the extent to which a product can be used by specified users to achieve specified goals with effectiveness, efficiency, and satisfaction in a specified context of use” [11]. Therefore, understanding users, their tasks, and their context is central to designing digital solutions for usability [12-14], as has been demonstrated in the design of some mHealth solutions in LMICs [15-20].

Studies investigating the use of mHealth solutions have found that factors relevant to technical artifacts, the environment a system is deployed in, and user characteristics have a significant impact on system use. Environmental factors with a bearing on mHealth use include those pertaining to the wider social, political, and economic context in which the solutions are deployed [18,19,21-27], as well as interactions among users and the leadership of the health care system [24,28,29]. User characteristics such as motivation, experience, and technological competence also affect mHealth use [15,17,21-23,30-32]. Further, technological factors relating to the artifacts of the solution, such as cost, user interface, and battery capacity, also have a significant impact on how solutions are used [15,19,25,26,29,33].

Given the variety of factors affecting mHealth use in LMICs, human-centered design processes for these systems require comprehensive explorations with end users to examine a priori user interactions with the intervention in the environment in which it will eventually be deployed. Human-centered design methods aim to ascertain users’ desires, needs, and experiences in order to design intuitive systems [34]. Human-centered design draws on several techniques, including but not limited to FGDs (focus group discussions), contextual interviews, participant observation, prototyping, and usability testing [35].

Qualitative research methods allow for such explorations and have been used to examine mHealth solutions [36,37], study human-computer interaction [38], and develop personas used to guide decision-making during design [39]. Consequently, qualitative research has been used in the design of mHealth solutions for health systems [15,16,18,20,40,41].

There is no well-established mechanism for exploring health care workers’ perceptions of and experiences with information systems deployed in LMIC health systems. We therefore developed a tool for guiding FGDs and key informant interviews with end users while optimizing an mHealth solution for a national public health program in India. The Vinyasa Tool is relevant to designers, developers, implementers, and health system administrators seeking to optimize mHealth systems or digitize health information systems, particularly in LMICs. We describe here the development of the Vinyasa Tool through the application of the Informatics Stack framework [42] to assimilate findings from literature.

## Methodology: Development of the Vinyasa Tool

### Reviewing Existing Frameworks for mHealth Research

We first describe the key frameworks that have guided inquiry into mHealth solutions in LMIC settings. We searched the literature on electronic health (eHealth) and mHealth systems in LMICs to identify frameworks developed and used to model these systems or to study their use by health care workers.

Chattopadhyay [43] developed a framework for studying health care staff perceptions regarding eHealth and organizational information communication technology (ICT) support for eHealth among primary health centers in India. Factors affecting user perceptions of eHealth were grouped into personal constructs regarding users’ levels of technology adoption and attitudes toward technology and organizational constructs that accounted for technology availability at the health centers.

Subsequently, Labrique et al [44] developed the mHealth and Information Communication Technology (ICT) Framework to describe mHealth solutions for strengthening reproductive, maternal, newborn, and child health in health systems. The framework depicts mHealth solutions in terms of the ICT applications used to address specific health system constraints along the reproductive, maternal, newborn, and child health continuum of care, the points of contact between the beneficiaries, providers, and facilities using the solution, and the timing of these contacts.

Vedanthan et al [45] categorized their findings from users of an integrated decision support and electronic health record tool, the DESIRE (Decision Support and Integrated Record-Keeping) tool, for hypertension in western Kenya. They categorized the barriers to implementation they elicited and their proposed solutions into 2 groups: a technical axis and a human axis. The technical axis consists of barriers in the server, cellular network, hardware, software, and user interface and design. The human axis consists of barriers in program administration, programmers and IT support staff, clinical mentors, nurses and clinical staff, and patients.

Mwendwa [31] used the Task Technology Fit model [46] to develop a conceptual framework for a study among community health workers using an mHealth solution (the RapidSMS solution) for maternal and child health in Rwanda. The framework was used to assess the ability of the solution to support the tasks of health workers. The questions of the focus group discussion guide and findings were categorized by the concepts of the framework: contextual fit, user comfort fit, workload fit, information communication fit, location fit, time criticality fit, and interaction fit.

Maar et al [47] developed a framework for process evaluations of mHealth solutions. It consists of process evaluation domains based on human organizational levels: patients, providers, community, and health system or setting. They applied the framework to the DREAM-GLOBAL (Diagnosing Hypertension-Engaging Action and Management in Getting Lower Blood Pressure in Indigenous and Low- and Middle-Income Countries) trial, which investigated an mHealth intervention to support control of hypertension in multiple low-resource settings.

More recently, Abejirinde et al [21] developed a model of the interactions between mHealth solutions and social and health systems in producing outcomes for maternal health in LMICs from a realist review of the literature. A framework was developed by extending the concepts of realist reviews to the “intervention, context, actor, mechanism, and outcome” framework. This framework considers a broad range of factors, including contextual factors, technological factors, user characteristics, and human-computer interaction. In their model, the interactions between these factors determine mHealth use and, subsequently, health outcomes. The investigators carried out a realist review of the literature, extracting data and identifying mechanisms underlying the results found. They determined the configurations of the concepts and populated the framework using the findings of their review. The framework produced describes how mHealth affects health outcomes and can be adapted for mHealth solution design.

The approaches to conceptualizing and depicting mHealth solutions described above successfully capture the environmental, user, and technological factors that affect mHealth solution use. The frameworks described do not explicitly address issues regarding the data, information, and knowledge (DIK) attributes of a system. Per the “Support Tool to Strengthen Health Information Systems: Guidance for Health Information System Assessment and Strategy Development” document from the World Health Organization (WHO), however, DIK is quite central to health information systems design [48]. The importance of end users’ perceptions about DIK finds empirical support from eHealth research in LMICs and theoretical explanation from information science. Studies among health workers in LMICs, ranging from community health workers [19,49] to district-level health authorities [49], have elicited perceptions regarding DIK and its effect on satisfaction with mHealth systems. The DeLone and McLean model of information system success links user information satisfaction to system use and consequently to the net benefits derived from a system [50]. To examine issues such as perceived redundancy of data entry or the perceived usefulness of the information produced, an exploratory tool would also need to incorporate DIK attributes in addition to those described in the frameworks above.

Lehmann [42] developed the Informatics Stack (the Stack) as a heuristic tool to facilitate a systems perspective on health IT systems. Although it was not conceptualized specifically for LMICs or mHealth solutions, its constructs and interrelationships are applicable to any setting and technology platform. The Stack envisages health IT solutions as a series of levels that envelop each other. Levels related to the environment of a solution encompass user-related levels, which themselves encompass levels related to the artifacts of a solution (including its DIK) [42]. Among other frameworks for studying health IT solutions, the “New Sociotechnical Model for Studying Health Information Technology in Complex Adaptive Health Care Systems” by Sittig and Singh [51] shares some components with the Stack, including DIK. However, Sittig and Singh’s model does not address the effects of privacy, security, interoperability, and the socioeconomic context of the environment on system use.

We build on the extensive body of work in the frameworks described above and choose the Informatics Stack as a framework we advance to incorporate the DIK attributes, data governance, and environmental factors of a health IT solution. Our tool consists of a list of questions with which to explore end users’ perceptions and experiences of mHealth systems, organized by the levels of the Stack. The tool includes inquiries into the wider contextual issues, user characteristics, human-computer interaction, and technological features that the other frameworks described address. We next describe the Informatics Stack in some detail, followed by our adaptation.

### The Informatics Stack

**Figure 1:**
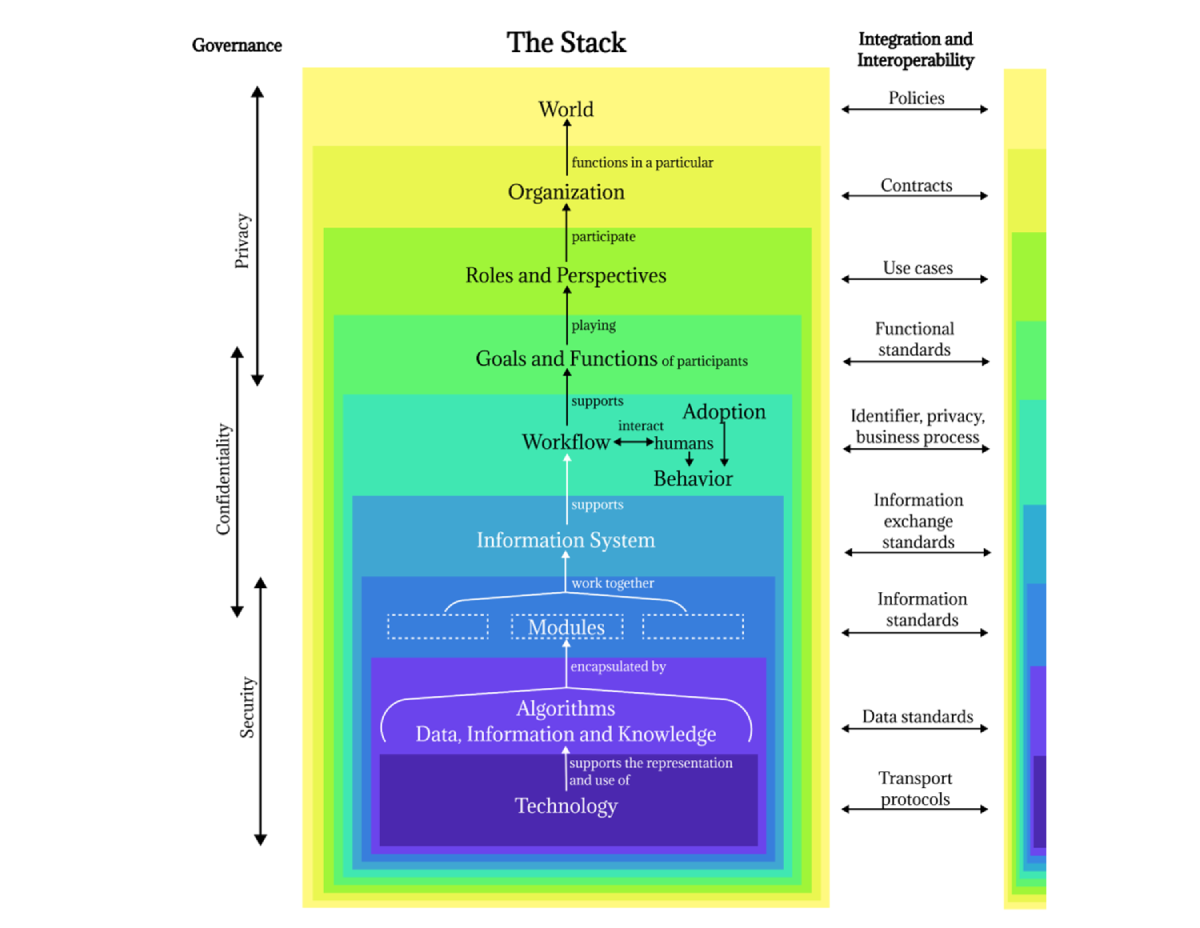
The concepts of the Informatics Stack adapted from Lehmann [42].

The Stack consists of 9 levels at which to view the components, interactions, governance, and interoperability of a health IT solution [42]. Data, information, knowledge, and algorithms form a distinct level in the Stack. Each level encompasses succeeding levels within itself as they proceed from the general context of the world in which the solution exists to the health care organization in which it is deployed through varying levels of focus, ending with the hardware and software used in the solution. The levels from top to bottom are “World,” “Organization,” “Roles and Perspectives,” “Goals and Functions,” “Workflow, Behavior, and Adoption,” “Information System,” “Modules,” “Data, Information, Knowledge, or Algorithms,” and “Technology.” The components of the Stack and interrelationships between the levels are illustrated in Figure 1, which is a representation of concepts from Lehmann’s [42] explanation of the Stack and adapted from his diagram.

We used the Stack to abstract the complex structures and interactions of mHealth solutions into simple sections to guide our inquiry with end users. We organized themes and findings from our literature review by the levels of the Stack, compiling a structured list of topics and questions, constituting the Vinyasa Tool.

We deviated from the Stack as developed by Lehmann [42] in 2 significant ways. First, governance (security, confidentiality, and privacy) and interoperability span multiple levels of the health informatics stack framework and are not themselves levels of the Stack. We collapsed governance and interoperability into new Stack levels in the Vinyasa Tool to group related themes for easier recall.

Second, we did not include the Stack level “modules” in our tool, as the subsystems conceptualized by Lehmann [42] do not find corollaries in mHealth literature investigating system use.

Using the Stack framework with the adaptations described, we assimilated themes and findings from a review of literature to populate the Vinyasa Tool as described below.

### Incorporating Themes Into the Vinyasa Tool

**Figure 2:**
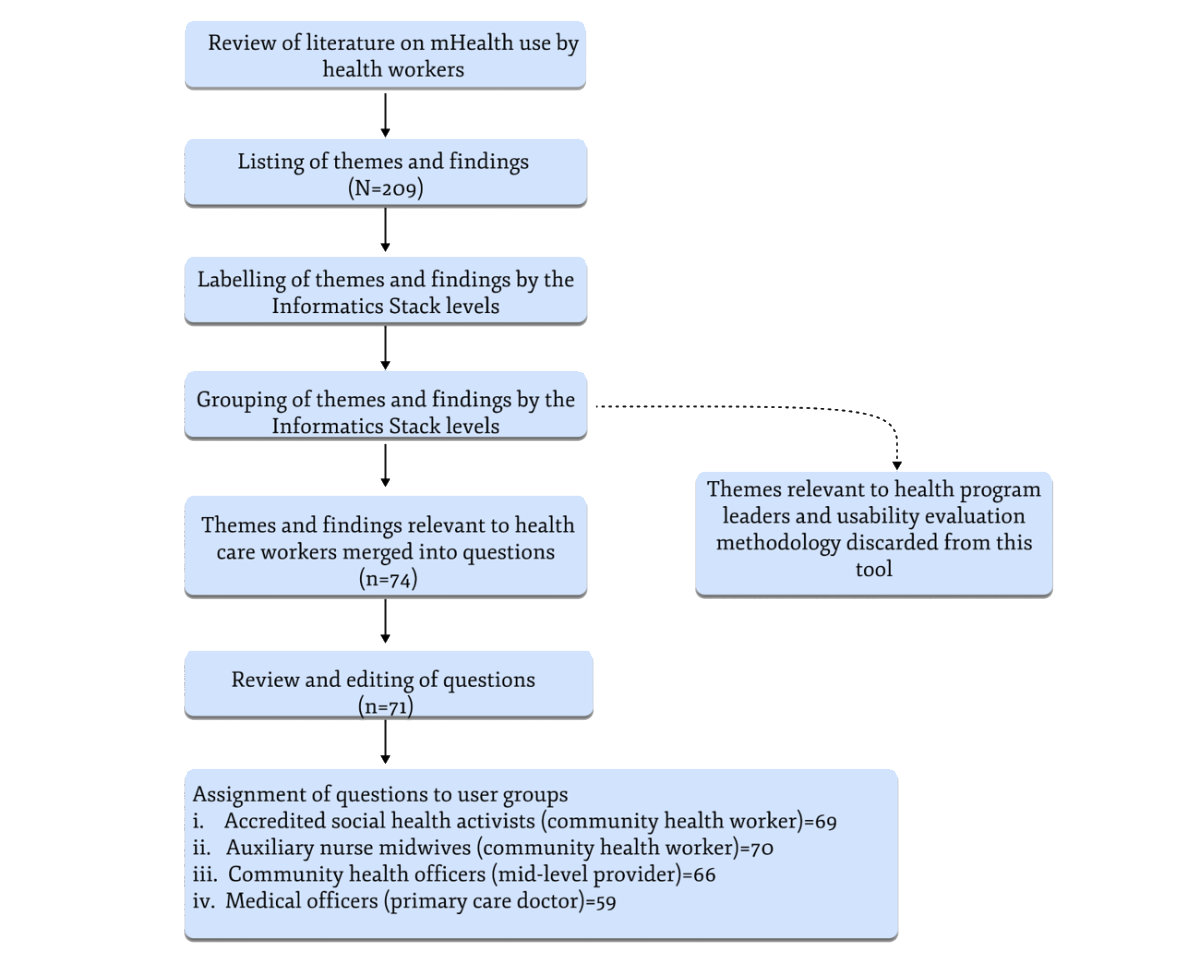
Identifying and incorporating themes into the Vinyasa Tool.

We conducted a review of the literature on mHealth use by health workers in LMICs to identify findings and themes relevant to health worker perceptions and experiences. The resulting themes and findings were used to form the questions that populated the Vinyasa Tool, as summarized in Figure 2 and described in steps 1 to 4 below.

1. We reviewed literature on theories of adoption and use of technology [52] and literature on mHealth use by health workers [17,40,53]. The search strategy included the terms *mHealth*, *health worker*, and *frontline worker*. We searched the Medline database for articles in English from 2015 to 2020.
2. Of these, 2 studies reviewed 82 articles from 2000 to 2018. Agarwal et al [53] present a systematic review of studies published in English between 2000 and 2013 on the feasibility and effectiveness of mHealth use by health workers in developing countries. The authors identified 1262 articles from an electronic database search and included 42 studies in a qualitative synthesis. The synthesis included peer-reviewed research articles and institutional reports with relevant terms for mHealth (defined as the use of mobile phones, tablets, personal digital assistants, and other hand-held wireless devices for health) and frontline health workers (health care personnel providing primary health care services in the community or clinics located in communities—community health workers, midwives, doctors, nurses, and pharmacists). Odendaal et al [40] present a qualitative evidence synthesis of 43 studies of mHealth use by health workers, 40 of which were not included in the qualitative synthesis by Agarwal et al [53]. They identified 7381 records from searching databases and gray literature without language, geographical, or date restrictions (until 2018). They selected 53 studies for inclusion in the review and 43 studies for a qualitative synthesis. Studies included in the review investigated the perceptions and experiences of persons providing primary health care services or supporting the provision of primary health care services in using mHealth technologies (defined as mobile devices used to create, store, retrieve, and transmit data in real time between users). We compiled a list of 209 themes from the 88 studies summarized in the articles reviewed and questions we considered important based on our experience in public health informatics.
3. The listed themes were mapped to Stack levels and sublevels as described in Table 1.
4. Through an iterative process of clustering and collapsing semantically similar themes, we finalized a list of 71 questions as described in Figure 2.

**Table 1:** Stack levels and examples of mapped themes.

Stack level	Examples of themes mapped to the Stack level
World	The effects of gender discrimination, transport availability, and language differences on mHealtha solution use [24,54] Community acceptance of the solution [51]
Organization	Health worker supervision through the mHealth solution [55] Communication with peers and supervisors [25,30]
Roles and Perspectives	The fit between the solution and the working roles of the users [19,31]
Goals and Functions	Changes in time spent with patients [51] Changes in relationships with the community [56] Effect on organizing work [19,34]
Workflow, Behavior, and Adoption	Effect of the solution on the users’ workloads [33,57] Integration of the solution into the user’s routine workflow [48] Effect of users’ digital literacy on solution use and effectiveness [28,55,58] Perceptions and experiences of training for the mHealth solution [29,34,44]
Information System	Comparisons with paper-based information systems [59]
Privacy, Security, and Confidentiality	Concerns of patients and the wider community regarding the confidentiality of information recorded on the device [25]
Interoperability	Duplication of data entry in multiple systems [33]
Data, Information, Knowledge, and Algorithms	Perceptions about the clinical decision support given by the solution [57] Satisfaction with the reports produced [60]
Technology	Concerns about the loss or damage of equipment [53] Preference for mobile phones or tablets [35]

^a^mHealth: mobile health.

## The Vinyasa Tool

**Figure 3:**
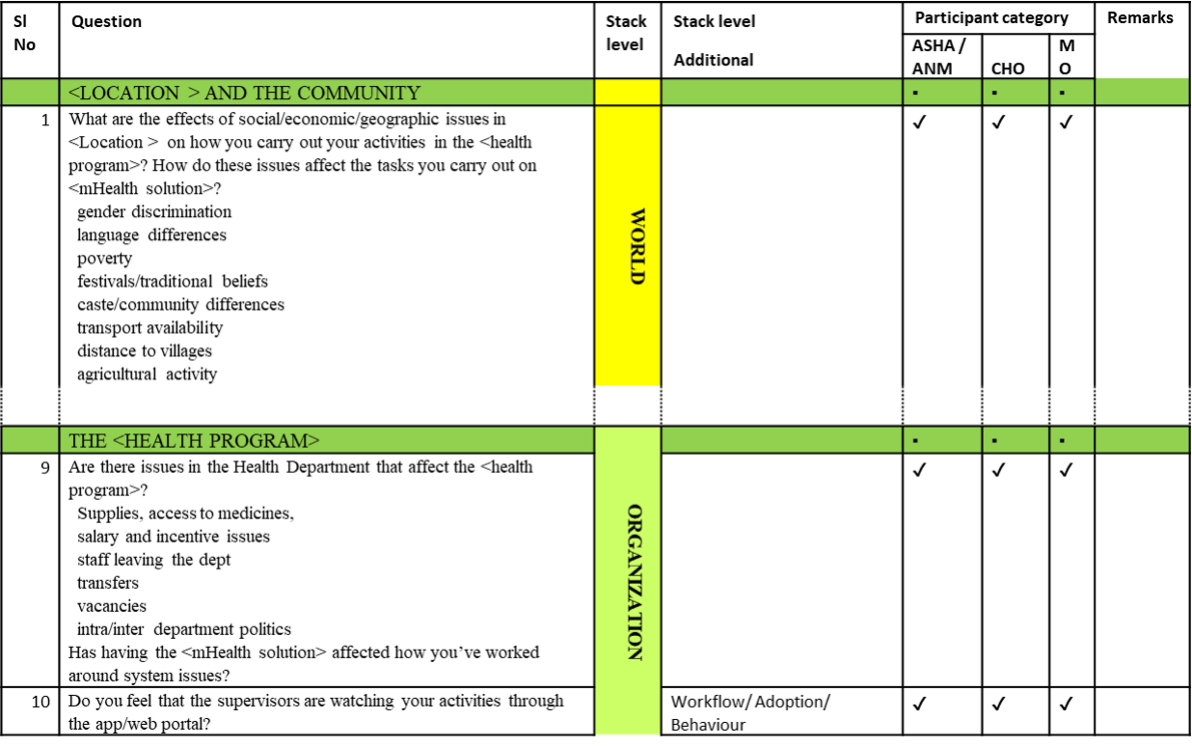
The Vinyasa Tool. SI: serial; ANM: auxiliary nurse midwife; ASHA: accredited social health activist; CHO: community health officer; mHealth: mobile health; MO: medical officer.

The Vinyasa Tool comprises 71 questions, grouped by the levels of the Stack and arranged as a table as illustrated in Figure 3. Each row is a question with prompts listed for specific areas of inquiry. Each question has an additional Stack level column if the question is relevant to more than one level of the Stack. This is followed by the participant categories to which a question is assigned. The tool has been designed for use by designers and developers to understand the needs and workflows of mHealth system end users in primary health care—community health workers, mid-level providers, and primary care physicians in LMIC settings. In India, these end users correspond to accredited social health activists—village-level community health workers [61], auxiliary nurse midwives—community health nurse midwives [61], community health officers—a cadre of mid-level health providers [62], and medical officers—graduate medical doctors based in primary or secondary health centers [63]. All of them have mixed clinical and public health responsibilities; some of them are responsible for program implementation and monitoring, as well.

## Using the Vinyasa Tool

The Vinyasa Tool aims to facilitate explorations with research study participants in either FGDs or key informant interviews. The tool covers a broad range of issues relevant to users of mHealth for primary health care, particularly in LMIC settings.

We conducted FGDs and contextual interviews with community health workers, mid-level providers, and primary care physicians in Government of Karnataka health facilities in rural areas of Mysore District between August and November 2021. The findings from the FGDs and contextual interviews were triangulated with findings from other human-centered design research methods, including heuristic evaluations, participant observations, workflow mapping, and usability tests. The composite findings from these research activities will be reported elsewhere. We report here our experience with using the Vinyasa Tool as a case study in Textbox 1.

Our application of the Informatics Stack to develop a tool for qualitative investigation of an mHealth solution in India is a novel contribution to qualitative research to improve mHealth. It enables a comprehensive and flexible exploration of health care workers’ perceptions and experiences of mHealth solutions. Its use would support human-centered design of mHealth solutions for LMICs, resulting in better mHealth use and the strengthening of health systems.

**Figure 4:**
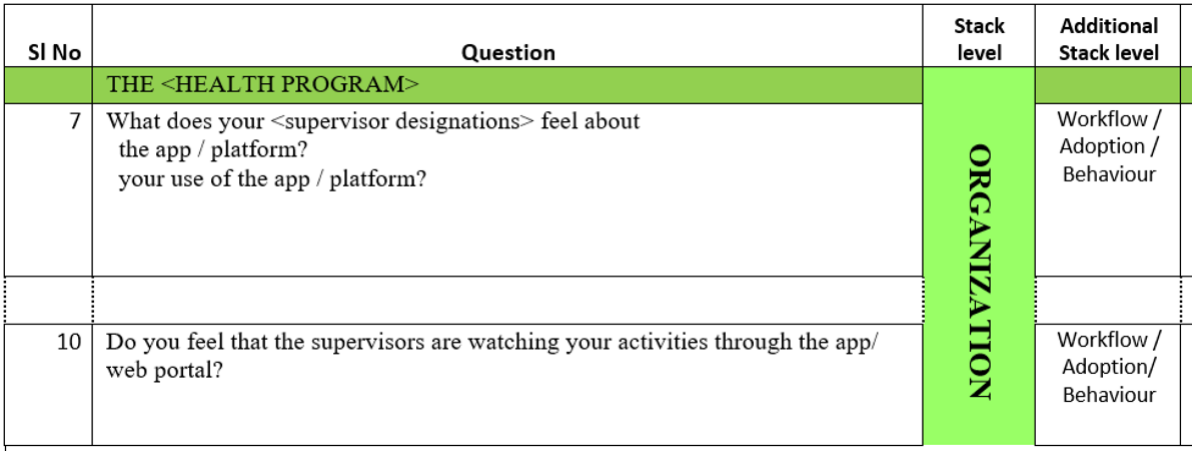
Examples of additional Stack levels in the Vinyasa Tool. SI: serial.

It is important to keep in mind that the Stack is a heuristic—a rule of thumb. We have used it as a method for abstracting the complex architectures of health informatics solutions into simpler sections for guiding our inquiries with users. The tool is best used if it is not considered a rigid structural schematic. We expect disagreement over the Stack levels to which we have assigned specific questions or themes because the research findings we used to populate the tool are not necessarily exclusive to individual Stack levels. Rather, some findings overlap across adjacent levels depending on the solution under investigation or the perspective of an investigator. For this reason, we included a column for additional Stack levels for each question to provide flexibility and keep the tool suitable for a variety of solutions and contexts. For example, while addressing work supervision of health workers, questions are in the Organization Stack level of the tool but are also labeled with the Workflow, Behavior, and Adoption Stack level in the additional Stack level column, as illustrated in Figure 4. This is because perceptions regarding supervisors have a significant effect on adoption and system use behavior.

We developed the Vinyasa Tool for the purpose of studying an existing mHealth solution to aid optimization. The approach we used can be adapted to the development of a digital solution to replace an existing paper-based health information system because the Stack levels are applicable to information systems regardless of the nature of the technology used. We suggest the tool be used in community and primary care settings in LMICs to assess the needs and workflows of physician and nonphysician clinical health workers. We are currently using the tool to optimize the implementation of the Indian government’s noncommunicable disease screening and management tool, targeting 100 million households in India. The Vinyasa Tool can be accessed in Multimedia Appendix 1 for use and adaptation.

**Using the Vinyasa Tool: a case study.**

**Introduction**

We used the Vinyasa Tool to explore the perceptions and experiences of end users of the Comprehensive Primary Health Care Non-Communicable Disease (CPHC NCD) solution in the state of Karnataka, India. The CPHC NCD solution is a national screening and management tool deployed in primary health care systems across India. It supports surveillance and continuity of care for diabetes mellitus, hypertension, oral cancer, breast cancer, and cervical cancer. By 2018, the system had enrolled over 100 million beneficiaries [64] and is currently among the largest digital health platforms anywhere in the world. This study was undertaken to optimize the usability of the CPHC NCD solution using the principles of human-centered design.

**Methodology**

We conducted 8 focus group discussions (FGDs) with 31 participants in total and 31 contextual interviews with community health workers, mid-level providers, and primary care physicians in Government of Karnataka health facilities in rural areas of Mysore District between August and November 2021. We elicited facilitators, barriers, suggestions, and requests from the participants using the solution. The findings from the FGDs and contextual interviews were triangulated with findings from other human-centered design research methods, including heuristic evaluations, participant observations, workflow mapping, and usability tests. This study was approved by the Institutional Ethics Committee of St. John’s Medical College and Hospital, Bangalore (Ref No 237/2019), and informed consent was obtained from all participants.

**Our experience using the Vinyasa Tool**

- We found the tool useful to be a comprehensive but flexible guide for our explorations with users. The tool enabled us to explore a wide range of topics related to the mHealth system being studied. The flow of our discussions jumped spontaneously between multiple diverse but interrelated issues, reflecting the sociotechnical nature of mHealth systems. We were able to keep track of these conversations and guide them using the Vinyasa Tool. The tool also enabled us to organize our observations and findings at the time of data collection to aid recall during subsequent FGDs or interviews for comparison with previous findings.
- We were able to limit our FGDs to 2 hours (including pile sorting and rating exercises) and contextual interviews to 1.5 hours, keeping within the limits of time acceptable for these sessions [65]. Some topics of the tool were covered spontaneously during the discussions without the questions in the tool being asked and others were not relevant to health workers who were unfamiliar with the mHealth solution. This allowed us to finish our FGDs and key informant interviews within the allotted time.

## Limitations

Our tool has some limitations, the chief among which is its length. While we believe it is comprehensive, it requires time and rigor to implement. Developers may benefit from reviewing the tool in its entirety and using components that they want to explore in further depth with their intended users, as they may have already addressed some of the challenges the tool seeks to highlight. Second, the tool was developed in the particular context of primary care settings in India. However, attempts to digitize primary care delivery in most LMIC settings are not dissimilar, and the tool lends itself to easy contextualization in other settings.

## Conclusions

The Vinyasa Tool facilitates a comprehensive capture of users’ perspectives on mHealth solutions. The tool aids in the understanding of user practices, the evaluation of systems to fix usability issues, and the participation of users in design, all of which are necessary to optimizing health IT solutions [66]. As a tool developed and used in India, the Vinyasa Tool has the potential to be useful for exploring mHealth solutions with Indian health care workers and others in similar contexts elsewhere. A failure to incorporate such perspectives into mHealth system design often results in persistent failure to meet system objectives. When the realities of health systems are not considered during the design phase, the ensuing design-reality gap becomes irreconcilable, leading to poor system use and system failure [67]. Such failures nullify the impact of the tremendous investments being made in mHealth in India and other LMICs.

The dearth of tools to support human-centered design of mHealth solutions and their implementation in LMICs is indicative of the absence of design considerations in LMIC health information system development [68]. While human-centered design has been established and mainstreamed into the design of commercial mobile applications the world over, its absence from digital health innovation in low-resource settings is stark. There is an urgent need among digital health stakeholders in India and other LMICs to institutionalize the principles and processes of human-centered design, ensuring that resources allocated for digital health deliver the benefits expected from them. In addition to human-centered design, other strategies necessary for the success of health information systems that need to be acknowledged include providing effective training [69] and ensuring continued technical support for users [70].

By applying the Informatics Stack framework [1] to key findings from the literature, we developed a tool that facilitates a comprehensive inquiry of the perceptions and experiences of health care workers using mHealth systems. The tool can be used to guide FGDs and contextual interviews with health care workers using mHealth solutions in India and similar countries. The Vinyasa Tool has the potential to contribute to the strengthening of the health care system by optimizing mHealth solutions for successful uptake in LMICs. The Vinyasa Tool is available in Multimedia Appendix 1 and on the website of the India Digital Health Net [71], a collaborative of domain experts and policy makers committed to advancing the science and practice of digital health implementation in India. We are available through the India Digital Health Net to guide stakeholders such as digital health incubators, governments, hospital systems, or any other organization in using the tool to better align mHealth solution design with ground realities.

## Appendix

Multimedia Appendix 1 The Vinyasa Tool.

## Abbreviations

DESIRE: Decision Support and Integrated Record-Keeping
DIK: data, information, and knowledge
DREAM-GLOBAL: Diagnosing Hypertension-Engaging Action and Management in Getting Lower Blood Pressure in Indigenous and Low- and Middle-Income Countries
eHealth: electronic health
FGD: focus group discussion
ICT: information communication technology
LMIC: low- and middle-income country
mHealth: mobile health
WHO: World Health Organization
