# Supplementary material for: The Vinyasa Tool for mHealth Solutions: Supporting Human-Centered Design in Nascent Digital Health Ecosystems
Source: JMIR Form Res. 2023 Oct 2;7:e45250. doi: 10.2196/45250 (PMC10580130; doi:10.2196/45250)
Supplement: Multimedia Appendix 1 [file formative_v7i1e45250_app1.docx]

**VINYASA TOOL St John’s Research Institute, Bangalore**

| **Sl. No.** | **Question** | **Stack level** | **Additional Stack level** | **ASHA /ANM** | **CHO** | **MO** | **Remarks** |
| --- | --- | --- | --- | --- | --- | --- | --- |
|  | <LOCATION>AND THE COMMUNITY | **WORLD** |  |  |  |  |  |
| 1 | Perceptions of the community about the app / devices?   - Reliability - Security of information - Prestige - Novelty - Any other aspect   Did COVID have any effect?  Has the device changed their interactions/ confidence/ trust in you? |  |  | ✓ | ✓ | ✓ |  |
| 2 | Have perceptions of technology (apps/ tabs/ mobile phones etc) changed in the community over time? |  |  | ✓ | ✓ | ✓ |  |
| 3 | What is the opinion of Thought Leaders in the community about the device / app? |  |  | 🗶 | 🗶 | ✓ |  |
| 4 | What is the effect of devices on your social status among the community and the perception of your own families about you? |  | Perspectives/ Roles  Technology | ✓ | ✓ | 🗶 |  |
| 5 | Do patients have feelings of discomfort during the use of <mHealth solution>? |  | Workflow/ Adoption/ Behavior | ✓ | ✓ | ✓ |  |
| **Sl. No.** | **Question** | **Stack level** | **Additional Stack level** | **ASHA /ANM** | **CHO** | **MO** | **Remarks** |
| 6 | What are the effects of social/ economic/ geographic issues in <Location> on how you carry out your activities in the <Health Program>? How do these issues affect the tasks you carry out on the app/ portal?   - gender discrimination - language differences - poverty - festivals - traditional beliefs - caste/ community differences - transport availability - distance to villages - agricultural activity | **WORLD** |  | ✓ | ✓ | ✓ |  |
|  | THE <HEALTH PROGRAM> | **ORGANIZATION** |  |  |  |  |  |
| 7 | What does your <supervisor designations> feel about   the app / platform?  your use of the app / platform? |  | Workflow / Adoption / Behavior | ✓ | ✓ | ✓ |  |
| 8 | Does the algorithm fulfil the requirements of the roles of the health workers and objectives of the program? |  | Perspectives/ Roles    Goals / Functions | 🗶 | 🗶 | ✓ |  |
| 9 | Are there issues in the Health Department that affect the <HEALTH PROGRAM>program? Supplies, access to medicines, salary and incentive issues staff leaving the dept transfers vacancies intra/ inter department politicsHas having the tool affected how you've worked around system issues? |  |  | ✓ | ✓ | ✓ |  |
| **Sl. No.** | **Question** | **Stack level** | **Additional Stack level** | **ASHA /ANM** | **CHO** | **MO** | **Remarks** |
| 10 | Do you feel that the supervisors are watching your activities through the app/ web portal? | **ORGANIZATION** | Workflow / Adoption/ Behavior | ✓ | ✓ | ✓ |  |
| 11 | How do you feel about the automated messages   are they helpful/ not helpful?   do you like them/ not like them?  does it feel like supervision?   does it feel supportive?   do you have guilt over not completing any tasks? |  | Workflow/ Adoption/ Behavior | ✓ | ✓ | 🗶 |  |
| 12 | Do you ever fear that the <MHEALTH SOLUTION>tool could become so efficient it could replace HCWs? |  | Workflow/ Adoption/ Behavior | ✓ | ✓ | 🗶 |  |
|  | ROLES/ PERSPECTIVES |  |  |  |  |  |  |
| 13 | What are your roles as ASHAs/ ANMs/ MLHPs/ MOs in general and in the <Health Program>? How can the <mHealth solution> help you fulfil your roles better? |  |  | ✓ | ✓ | ✓ |  |
| 14 | What support from your leadership do you have for using the <MHEALTH SOLUTION>tool?  How do the program leaders influence your use of the <mHealth solution>?   What should their influence be like? |  | Organization  **ROLES/**  **PERSPECTIVES** | ✓ | ✓ | ✓ |  |
| 15 | Does the <mHealth solution> help you supervise health workers reporting to you?  if yes, how? if no, how can it? |  | Organization  DIKW / Algorithm | 🗶 ✓ | ✓ | ✓ |  |
| **Sl. No.** | **Question** | **Stack level** | **Additional Stack level** | **ASHA /ANM** | **CHO** | **MO** | **Remarks** |
|  | EFFECT of <MHEALTH SOLUTION> ON GOALS/ FUNCTIONING: Community/ Patient Interaction | **GOALS / FUNCTIONING** |  |  |  |  |  |
| 16 | How have your interactions with the community/ patients changed while using the <MHEALTH SOLUTION> app/ other data collection tools – duration, ease of listening, rapport building |  |  | ✓ | ✓ | 🗶 |  |
| 17 | Has the <mHealth solution> changed how well you know your patients in the community – has it made things better/ worse? Why did things change? |  |  | ✓ | ✓ | ✓ |  |
| 18 | Has the <mHealth solution> changed how well you know the status of the community as a whole? |  |  | ✓ | ✓ | ✓ |  |
| 19 | Has the <mHealth solution> changed the amount of time spent with patients? |  |  | ✓ | ✓ | ✓ |  |
| 20 | How have your interactions with the patients changed while using the <MHEALTH SOLUTION> web portal? |  |  | 🗶 | 🗶 | ✓ |  |
|  | EFFECT of <MHEALTH SOLUTION> ON GOALS/ FUNCTIONING: Effect on health care delivery |  |  |  |  |  |  |
| 21 | Are there any medical procedures required through the <mHealth solution> which you feel are difficult to do?  If so, which? |  |  | ✓ | ✓ | ✓ |  |
| 22 | What are the challenges you are facing in screening and managing and any other aspect of the <Health Program>?  How can the tool help? |  |  | ✓ | ✓ | 🗶 |  |
| 23 | Is the <mHealth solution> making things easier/ harder or faster/ slower to get through your work?  Screening/ tracking/ monitoring/ follow up |  | Workflow/ Adoption/ Behavior | ✓ | ✓ | ✓ |  |
| **Sl. No.** | **Question** | **Stack level** | **Additional Stack level** | **ASHA /ANM** | **CHO** | **MO** | **Remarks** |
| 24 | Did the <mHealth solution> (app and tablets/ portal and PC) enhance your performance?  **GOALS / FUNCTIONING**   - Completion time - Organising work - Better information and tracking - Clinical work diagnosing and prescribing etc - Decision making - Better reporting |  | Workflow/ Adoption/ Behavior | ✓ | ✓ | ✓ |  |
| 25 | Explore the complicated cases the users have come across:   - What are the cases and how did the community feel about the condition? - Were there aspects of the app that helped with the difficult cases? - What's working/ what's missing/ what's beyond scope of the app/ portal? |  | World  Workflow/ Adoption/ Behavior | ✓ | ✓ | ✓ |  |
| 26 | Do you use the internet for getting health information for patients?   what for? |  |  | ✓ | ✓ | ✓ |  |
| 27 | Has the app / portal made it easier to schedule follow up home visits?  how? |  |  | ✓ | ✓ | ✓ |  |
| 28 | Effect on flexibility of work schedule   - How has your day planning changed because of the <MHEALTH SOLUTION> app? - Do the different health program apps have different work schedules/ plans for each day i.e. different locations/ patients to be visited on the same day?  if yes, how do you manage doing different work schedules of the different programs? |  |  | ✓ | ✓ | 🗶 |  |
|  | EFFECT of <MHEALTH SOLUTION> ON GOALS/ FUNCTIONING: Effect on Health Workers |  |  |  |  |  |  |
| 29 | Has the <mHealth solution> had any effect on your travel time – to base hospital for reporting, or any other travel required for work? |  |  | ✓ | ✓ | 🗶 |  |
| **Sl. No.** | **Question** | **Stack level** | **Additional Stack level** | **ASHA /ANM** | **CHO** | **MO** | **Remarks** |
| 30 | Has the solution improved your access to updated medical information and access to professional advice and information?   how? | **GOALS / FUNCTIONING** |  | ✓ | ✓ | ✓ |  |
| 31 | Has the <mHealth solution> enabled better communication and coordation between you, other health care workers, supervisors or patients? If so, how? |  |  | ✓ | ✓ | ✓ |  |
| 32 | Has the <mHealth solution> affected your work load?   reactions  expectations for renumeration if any |  | Perspectives/ Roles | ✓ | 🗶 | 🗶 |  |
| 33 | Has the <mHealth solution> affected your incentives?   Difference with the paper based system? |  | Technology | ✓ | 🗶 | 🗶 |  |
| 34 | Effect on productivity  Are there distractions with the mobile/ misuse of the mobile?  Has it changed as you've had more time to use mobile devices? |  | Workflow/ Adoption/ Behavior | ✓ | ✓ | ✓ |  |
| 35 | Has using tablets affected how much weight/materials you must carry during work?   - more weight or less weight? |  |  | ✓ | ✓ | 🗶 |  |
| **Sl. No.** | **Question** | **Stack level** | **Additional Stack level** | **ASHA /ANM** | **CHO** | **MO** | **Remarks** |
|  | EXPERIENCE | **WORKFLOW / ADOPTION / BEHAVIOR** |  |  |  |  |  |
| 36 | What were your initial impressions/ experiences with EHRs/ mobile apps/ Digital health tools? |  |  | ✓ | ✓ | ✓ |  |
| 37 | What were your expectations of and feelings about the app / portal before you started using it?   - ease of learning - effect on work/ time required - expectation vs experience of solution |  |  | ✓ | ✓ | ✓ |  |
| 38 | What is your experience with computers, mobile phones for personal use and for work? What were the first devices and apps you used and what are you using now? Did previous experience with computer/ mobile devices help you:   - use the <MHEALTH SOLUTION> app / portal? - carry out tasks for community members/ patients using the app? - If yes - how? |  |  | ✓ | ✓ | ✓ |  |
|  | TRAINING and SUPPORT |  |  |  |  |  |  |
| 39 | What was the learning and training experience of the <mHealth solution>?   - Adequacy - Confidence |  |  | ✓ | ✓ | ✓ |  |
| **Sl. No.** | **Question** | **Stack level** | **Additional Stack level** | **ASHA /ANM** | **CHO** | **MO** | **Remarks** |
| 41 | How often do you have refresher training?   - Is it useful? - Does the effect last? - Current training needs - Do the other staff using the tool know the basics of mobile operation? | **WORKFLOW / ADOPTION / BEHAVIOR** |  | ✓ | ✓ | ✓ |  |
| 42 | What is the support system for the app and hardware?   - Who do you go to for help? - Are there resources to help you? - Are there local places for fixing devices/ app problems? - Time to resolution |  | Organization | ✓ | ✓ | ✓ |  |
| 43 | Is there a backup plan to use if the device/ app are not working?   - What happens to the data which was on the device before the breakdown? - What happens to data collected while the device is broken? |  |  | ✓ | ✓ | ✓ |  |
|  | WORKFLOW |  |  |  |  |  |  |
| 44 | How do you manage the data entry for the <MHEALTH SOLUTION> web portal at your PHC?   - personnel - frequency - timing   How does the data entry affect the flow of patients through your health centre?  Time   - queues - bottlenecks |  | Organization  Goals/ Functioning | ✓ | 🗶 | 🗶 |  |
| **Sl. No.** | **Question** | **Stack level** | **Additional Stack level** | **ASHA /ANM** | **CHO** | **MO** | **Remarks** |
| 45 | How do you use the <mHealth solution> for referring patients?   - decision to refer - preparing patient for referral communication - preparing to receive patients at your facility - tracking patients and follow up after return to home - reports of referrals | **WORKFLOW / ADOPTION / BEHAVIOR** |  | ✓ | ✓ | ✓ |  |
|  | ADOPTION/ SYSTEM USE BEHAVIOR |  |  |  |  |  |  |
| 46 | Do you feel that any of the tasks you have to do in the <MHEALTH SOLUTION> App/ Web portal are unnecessary, unimportant, "extra" to your main work? |  |  | ✓ | ✓ | ✓ |  |
| 47 | Has the <MHEALTH SOLUTION> App/ tool changed your view of the importance of data collection? |  |  | ✓ | ✓ | ✓ |  |
| 48 | How do you use the <MHEALTH SOLUTION> app/ web portal at your PHCs?   - clinical - public health activities |  |  | 🗶 | 🗶 | ✓ |  |
| 49 | How is the ease of use of the <MHEALTH SOLUTION> app / web portal?   - Ease of use - Time taken for data collection, screening, etc - Number of login credentials - Spelling errors - Navigation - finding your way through the app, - Knowing where you are while using the app |  | Technology | ✓ | ✓ | ✓ |  |
| 50 | Is it important for the <MHEALTH SOLUTION> app/ web portal to be easy to use?  If the app / tool is not easy to use, how does it affect you/ your work? |  |  | ✓ | ✓ | ✓ |  |
| **Sl. No.** | **Question** | **Stack level** | **Additional Stack level** | **ASHA /ANM** | **CHO** | **MO** | **Remarks** |
|  | INFORMATION SYSTEMS: Comparing the <mHealth solution> with other systems | **INFORMATION SYSTEM** |  |  |  |  |  |
| 51 | Compare <MHEALTH SOLUTION> app vs Other health program apps:   - Appearance - Ease of use - Effect on workflow - Reporting/ report generation - Data loss, - security and privacy |  | Organization  Workflow/ Adoption/ Behavior | ✓ | ✓ | ✓ |  |
| 52 | Advantages & disadvantages of the app compared to the previous data collection method, for you, community members and patients |  | World | ✓ | ✓ | ✓ |  |
| 51 | How do other mobile apps, including non-health program apps, affect your <Health Program> activities?   - effect on how well the device works - effect on how you manage patients using the <mHealth solution> - disruptions from having many different apps - apps for communication, information, and location services - have they helped in carrying out program activities?  if so, how? |  | World  Workflow/ Adoption/ Behavior | ✓ | ✓ | ✓ |  |
|  | INTEROPERABILITY |  |  |  |  |  |  |
| 52 | Do you have repeated data entry in the <MHEALTH SOLUTION> app and across different health program apps?  If so, what do you think about it?  How could the apps be improved regarding repeated data entry? |  | Technology  **INTER**  **OPERABILITY** | ✓ | ✓ | ✓ |  |
| 53 | Are you entering physical registers as well?  How much data is repeated in the app and the register? |  |  | ✓ | ✓ | ✓ |  |
| **Sl. No.** | **Question** | **Stack level** | **Additional Stack level** | **ASHA /ANM** | **CHO** | **MO** | **Remarks** |
|  | SECURITY and PRIVACY |  |  |  |  |  |  |
| 54 | Do you have concerns about how safe and private the information is? |  | **SECURITY**  **& PRIVACY** | ✓ | ✓ | ✓ |  |
| 55 | Do patients, family members and the wider community (e.g. community leaders) have any concerns over safety and privacy? |  | World | ✓ | ✓ | ✓ |  |
|  | DATA INFORMATION KNOWLEDGE / ALGORITHMS | **DATA INFORMATION KNOWLEDGE**  **ALGORITHMS** |  |  |  |  |  |
| 56 | How do you feel about recommendations from an app about:   - what diagnosis to make for a patient? - how to treat a patient? |  |  | ✓ | ✓ | ✓ |  |
| 57 | Are there any questions/ clinical signs/ sections in the <mHealth solution> which you feel are unnecessary for screening/ managing patients?   - which? - why?   Are there any questions/ clinical findings which should be added to the <mHealth solution>?   - which? - why? |  |  | ✓ | ✓ | ✓ |  |
| 58 | How do you feel about the reports and dashboard of the <mHealth solution>?   - Time saving - Completeness - Satisfaction with the reports/dashboard |  |  | ✓ | ✓ | ✓ |  |
| **Sl. No.** | **Question** | **Stack level** | **Additional Stack level** | **ASHA /ANM** | **CHO** | **MO** | **Remarks** |
|  | SOFTWARE | **TECHNOLOGY** |  |  |  |  |  |
| 59 | How is the appearance of the app / Web portal?   - Simplicity - Colour - Text consistency - Size - Icons etc |  |  | ✓ | ✓ | ✓ |  |
| 60 | Do you have any suggestions for how to make the app appearance or functioning better? |  |  | ✓ | ✓ | ✓ |  |
| 61 | Is the content appropriate for the <Health Program>?   - Headings - Search menu - Precise text - Terminology - Use of dropdowns/ checkboxes - Trouble shooting |  |  | ✓ | ✓ | ✓ |  |
| 62 | What would you like to be improved in the app? |  |  | ✓ | ✓ | ✓ |  |
|  | HARDWARE GENERAL |  |  |  |  |  |  |
| 63 | Are there concerns about equipment?   - Loss - Damage |  |  | ✓ | ✓ | ✓ |  |
| 64 | What are your impressions about the hardware (IT and non-IT including BP monitor etc.) you are using in the <Health Program>?   - Does it work well? - Do you find it easy to use? - Do you find it useful? |  |  | ✓ | ✓ | ✓ |  |
| 65 | What is your preferred type of device (Smartphone / Mobile vs Tablet) |  |  | ✓ | ✓ | 🗶 |  |
| 66 | Would you prefer to use your own device or use a device that belongs to the program? |  |  | ✓ | ✓ | 🗶 |  |
| **Sl. No.** | **Question** | **Stack level** | **Additional Stack level** | **ASHA /ANM** | **CHO** | **MO** | **Remarks** |
| 67 | Do you have any suggestions for hardware?   - casing/ protection - extra battery | **TECHNOLOGY** |  | ✓ | ✓ | 🗶 |  |
| 68 | Is access to phones/ devices access an issue in carrying out screening and management through <mHealth solution>? |  |  | ✓ | ✓ | 🗶 |  |
|  | NETWORK CONNECTIVITY |  |  |  |  |  |  |
| 69 | Are there network connection issues (at VILLAGE/ SC/ HWC/ PHC/ CHC) while using the app/ web portal?   - No internet connection areas - Loss of internet connection while using the app - Poor internet connection affecting syncing   If so, are there any back up measures? Does poor connection lead to patient dissatisfaction? |  | World | ✓ | ✓ | ✓ |  |
| 70 | Are there times when you have to use your own money to run <mHealth solution> / access the internet for the <mHealth solution>? If so, how do you feel about using your own money? |  | Workflow/ Adoption/ Behavior | ✓ | ✓ | ✓ |  |
|  | BATTERY |  |  |  |  |  |  |
| 71 | Are there battery charge concerns? If so, any back up measures? How long should the battery last? |  |  | ✓ | ✓ | ✓ |  |
